# *Lactobacillus acidophilus* JCM 1132 Strain and Its Mutant with Different Bacteriocin-Producing Behaviour Have Various In Situ Effects on the Gut Microbiota of Healthy Mice

**DOI:** 10.3390/microorganisms8010049

**Published:** 2019-12-25

**Authors:** Gang Wang, Yunxia Yu, Enriqueta Garcia-Gutierrez, Xing Jin, Yufeng He, Linlin Wang, Peijun Tian, Zhenmin Liu, Jianxin Zhao, Hao Zhang, Wei Chen

**Affiliations:** 1 State Key Laboratory of Food Science and Technology, Jiangnan University, Wuxi 214122, China; wanggang@jiangnan.edu.cn (G.W.);; 2 State Key Laboratory of Dairy Biotechnology, Shanghai Engineering Research Center of Dairy Biotechnology, Dairy Research Institute, Bright Dairy & Food Co., Ltd., Shanghai 200436, China; 3 Gut Microbes and Health Institute Strategic Programme, Quadram Institute Bioscience, Norwich NR4 7UQ, UK; 4 Food Bioscience Department, Teagasc Food Research Centre, P61 C996 Fermoy, Ireland; 5 School of Food Science and Technology, Jiangnan University, Wuxi 214122, China; 6 International Joint Research Laboratory for Probiotics, Jiangnan University, Wuxi 214122, China; 7 (Yangzhou) Institute of Food Biotechnology, Jiangnan University, Yangzhou 225004, China; 8 National Engineering Research Center for Functional Food, Jiangnan University, Wuxi 214122, China; 9 Beijing Innovation Centre of Food Nutrition and Human Health, Beijing Technology and Business University (BTBU), Beijing 100048, China

**Keywords:** *Lactobacillus acidophilus*, bacteriocin, short-chain fatty acids, gut microbiota, in situ effects

## Abstract

The production of bacteriocin is considered to be a probiotic trait of lactic acid bacteria (LAB). However, not all strains of LAB harbour bacteriocin genes, even within the same species. Moreover, the effects of bacteriocins on the host gut microbiota and on host physiological indicators are rarely studied. This study evaluated the effects of the bacteriocin-producing *Lactobacillus acidophilus* strain JCM1132 and its non-producing spontaneous mutant, *L. acidophilus* CCFM720, on the physiological statuses and gut microbiota of healthy mice. Mice that received the bacteriocin-producing strain JCM1132 exhibited reduced water and food intake. Furthermore, the administration of these strains induced significant changes in the compositional abundance of faecal microbiota at the phylum and genus levels, and some of these changes were more pronounced after one week of withdrawal. The effects of CCFM720 treatment on the gut microbiota seemed to favour the prevention of metabolic diseases to some extent. However, individuals that received JCM1132 treatment exhibited weaker inflammatory responses than those that received CCFM720 treatment. Our results indicate that treatment with bacteriocin-producing or non-producing strains can have different effects on the host. Accordingly, this trait should be considered in the applications of LAB.

## 1. Introduction

The gut microbiota are among the most important and active components of intestinal micro-ecology. Accordingly, this structure has recently become a research hotspot in various scientific fields. More than 1000 bacterial species are estimated to reside in the human intestine, and these organisms harbour up to 100 times as many genes as the human genome [1]. The gut microbiota plays a very important role in host health. First, the microbiota colonises the intestinal mucosa and thus constitutes a natural protective barrier over the host intestine [2]. Second, the microbiota digests food nutrients, particularly some non-digestible polysaccharides by the host, to provide energy to the host [3]. Third, the microbiota inhibits the invasion and infection of pathogenic microorganisms by competing for intestinal nutrients and niches, as well as secreting bacteriostatic and antimicrobial substances [4,5]. Fourth, the microbiota synthesises beneficial metabolites, such as short-chain fatty acids (SCFAs) [6], vitamin B12 and folic acid [7]. Finally, the microbiota regulates the host immune system and promotes the maturation of immune cells [8]. The composition of the gut microbiota is determined by the mutual adaptation of microorganisms and their hosts, and these interactions maintain the functional stability of the intestinal micro-ecological system [9]. This system also involves metabolites produced by the gut microbiota, including SCFAs such as acetate, propionate and butyrate [6]. These SCFAs can activate G protein-coupled receptors on the surfaces of host cells to regulate the growth, metabolism and functions of a variety of cells, including intestinal epithelial cells, adipocytes and leukocytes [10,11].

‘Lactic acid bacteria’ (LAB) refers to a group of bacteria that degrade carbohydrates to produce lactic acid, which has antimicrobial properties. Accordingly, humans have used LAB in the fermentation and preservation of foods and eaten for thousands of years. These traditional uses have led to the perception of most LAB as organisms generally regarded as safe (GRAS). Other studies have shown that some LAB species provide benefits to the host and are therefore considered probiotic. Most probiotic species exhibit resistance to acids, bile salts and enzymes in the gastrointestinal (GI) environment and can adhere to the intestinal lining to colonise the human GI tract [12,13].

Bacteriocins are small antimicrobial peptides produced by bacteria which may be subject to posttranslational modification. The production of bacteriocin is usually considered to be a probiotic trait. Studies have identified bacteriocin-producing LAB in the GI tracts of hosts [14,15,16]. However, very few studies have investigated whether the production of bacteriocins by probiotic strains in the GI tract affect host health [17,18,19], other than studies in which pathogenic bacteria were antagonised.

In this study, two strains of *Lactobacillus acidophilus* that share similar physiological characteristics but differ in bacteriocin production, JCM 1132 and CCFM 720, were investigated to assess their effects on the gut microbiota and metabolite composition in healthy mice. *L. acidophilus* CCFM 720 is a spontaneous mutant strain with no antimicrobial activity, obtained from the bacteriocin-producing strain *L. acidophilus* JCM 1132 after multiple passages. These two strains enabled the study of the different effects of phenotypically different strains of the same LAB species on the gut microbiota and metabolite composition.

## 2. Materials and Methods

### 2.1. Measurement of Antibacterial Activities and Spectra

All of the bacterial strains engaged in this study are listed in Table 1. The bacterial strains were maintained at −80 °C in MRS containing 30% glycerol.

The tested strains were cultured in MRS broth (pH 6.2) at 37 °C for 20 h with 2% inoculum. Indicator strains were cultured according to the conditions in Table 1 with the same inoculum. The tested strains were sub-cultivated twice and the activity in the supernatants was tested using a well-diffusion assay [20]. Briefly, cell-free supernatants (CFS) were collected by centrifugation (8000× *g*, 20 min, 4 °C) and adjusted to a pH of 6.00 ± 0.05 using 4 M NaOH prior to filtration through a sterilised nitrocellulose membrane (pore size: 0.22 µm). Oxford cups were placed on a plate containing a layer of prepared water agar and were filled with medium containing the indicator bacteria at a density of 10^6^–10^7^ CFU/mL. The Oxford cups were removed after the medium solidified [21]. A volume of 100 µL of CFS was added to each well and allowed to diffuse at 4 °C for 4 h. Next, the wells were incubated at 37 °C for 16–18 h and examined to detect the formation of a clear zone of inhibition around the wells.

### 2.2. Sensitivity of Antibacterial Substances in CFS to Catalase, Protease and Heat

To evaluate the sensitivity of antibacterial substances to catalase and protease, neutralised CFS from each strain was incubated for 2 h at 37 °C with the following: catalase (3000 U/mg, Sangon, Shanghai, China), pepsin (15,000 U/mg, Sangon, Shanghai, China), trypsin (250 U/mg, Sangon, Shanghai, China) and papain (6000 U/mg, Sangon, Shanghai, China). The enzymes were used at a final concentration of 1 mg/mL in 50 mmol sodium phosphate buffer (pH 7.00), except for pepsin (pH 2.00) [22,23]. Subsequently, the enzymes were inactivated by boiling at 100 °C for 10 min, after which the pH of each CFS was re-adjusted to 6.00. The antibacterial ability was determined according to the procedure described in Section 2.1.

To evaluate the sensitivity of antibacterial substances to heat, CFS from each strain was incubated under the following conditions: 60 °C for 10 min, 60 °C for 30 min, 60 °C for 1 h, 90 °C for 10 min, 90 °C for 30 min and 121 °C for 15 min. Subsequently, the pH of each CFS was re-adjusted to 6.00. The antibacterial ability was determined according to the procedure described in Section 2.1.

### 2.3. Biological Characteristics of the Two Tested Strains

#### 2.3.1. Growth Curve, Generation Time and Adhesion Assay

After three rounds of propagation, the tested strains were inoculated in MRS broth (pH 6.2) at 37 °C with 2% inoculum, then mixed and dispensed into 25 sterile test tubes for cultivation under appropriate conditions. Bacteria were counted every hour using the plate count method except during the exponential phase, wherein the procedure was repeated every 30 min (one tube per count). The number of viable bacteria at each time point was plotted, and the generation time was calculated using the growth curve [6] of the two tested strains, according to the formula:

$$G=\frac{\left(t_{2}{\;-\;t}_{1}\right)}{3.322\;\times \;\left({\mathrm{lgN}}_{2}{\;-\;\mathrm{lgN}}_{1}\right)}$$

where t1 and t2 represent time points 1 and 2 during the exponential phase, respectively, and N1 and N2 represent the numbers of viable bacteria at t1 and t2, respectively.

For adhesion assays, Caco-2 cells were seeded (4 × 10^5^ cells/mL) into six-well tissue culture plates with RPMI-1640 medium supplemented with 10% (*v*/*v*) fetal bovine serum (Gibco, Grand Island, NY, USA) and cultured at 37 °C in an incubator with 5% CO_2_ until monolayers of cells were formed. Monolayers were washed twice with PBS (pH 7.2) before adhesion assays. 2 mL bacteria suspension (10^8^ CFU/mL) in antibiotic-free cell culture medium were added to each well and plates were incubated for 2 h. After that, cells were washed twice with PBS (pH 7.2), fixed with methanol for 30 min and then Gram-stained for microscopic examination under an oil immersion lens. The adherence index was evaluated in 20 random microscopic fields of adhering bacteria per 100 cells. Adherence assays were performed in triplicate.

#### 2.3.2. Determination of the Tolerance of Two Strains to Simulated GI Tract Conditions

The resistance of the two strains to gastric acid and bile salts was measured as described by Zhai [24], with modification. Simulated gastric juice was prepared by suspending pepsin (15,000 U/mg, Sangon, Shanghai, China) in sterile saline (0.85% *w*/*v*, pH 3.0) to a concentration of 3 g/L. Simulated small intestinal juice was prepared by adding trypsin (250 U/mg, Sangon, Shanghai, China) and 0.3% bile salts (Oxoid, Basingstoke, Hants, UK) to sterile saline (0.85% *w*/*v*, pH 8.0) at a concentration of 1 g/L. Both juices were filtered through a sterilised nitrocellulose membrane (pore size: 0.22 µm). Cells of the tested bacterial strains were collected by centrifugation at 5500× *g* for 15 min and resuspended in simulated gastric juices before obtaining initial viable cell counts. After 3-h incubation at 37 °C, the cultures were centrifugated at 5500× *g* for 15 min and resuspended in simulated intestinal juices for 2 or 4 h. The tolerance of both strains to the simulated GI tract conditions was analysed by evaluating the Colony-Forming Units of each strain after successive incubations in two juices via the spread plate method.

### 2.4. Genome Sequencing and Assemblies

The genome sequences of *L. acidophilus* JCM 1132 and CCFM720 were determined by Majorbio (Shanghai, China) on the Illumina Hiseq×10 platform. A coverage of at least 100-fold was achieved. Raw data were assembled using SOAP de novo2 software with the default settings. Coding sequences (CDS) were predicted using Glimmer, GeneMarkS and Prodigal. Glimmer was generally applied to bacterial chromosomes, while GeneMarkS was applied to plasmids. Otherwise, tRNA genes were identified using tRNAscan-SE version 2.0, whilst rRNA genes were detected using Barrnap software (https://github.com/tseemann/barrnap). BLASTp and a combination of the Swiss-Prot, Pfam, EggNOG, GO, KEGG and NR (non-redundant) GenBank databases were used to perform the functional annotation. The genomes were analysed using BAGEL4 [25] for bacteriocin prediction and compared using Blast. Proteome comparison was performed using PATRIC V.3.5.43 with default parameters [26].

### 2.5. MALDI-TOF MS

50 µL of CFS of JCM 1132 or CCFM720 were mixed with 50 µL of 70% propan-2-ol with 0.1% trifluoroacetic acid and analyzed by MALDI-TOF mass spectrometer (Bruker Daltonics Inc., Billerica, MA, USA) to determine the masses of the potential peptides [27]. Results were analyzed using FlexAnalysis V3.4 (Bruker Daltonics Inc., USA) software.

### 2.6. Animals and Experimental Design

**Figure 1:**
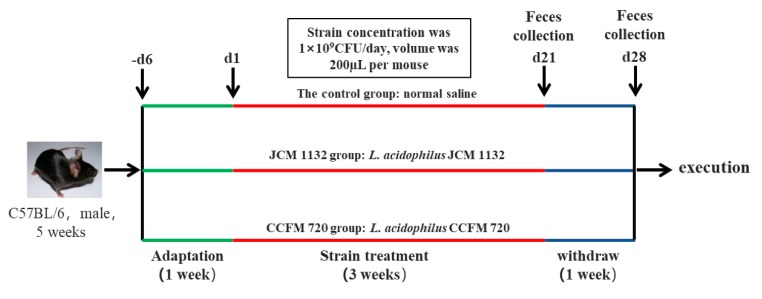
Animal experimental design. Eighteen mice were randomly and equally divided into three groups: control, *L. acidophilus* JCM 1132 and CCFM 720. After adaptation, the mice in the control group were gavaged with normal saline for 3 weeks. The mice in the JCM 1132 and CCFM 720 groups were respectively gavaged with 1 × 10^9^ CFU of JCM 1132 or CCFM 720 per mouse for 3 weeks. For all groups, gavage was then withdrawn for 1 week. Faeces were collected after the 3-week gavage period and 1-week withdrawal period (days 21 and 28, respectively).

Five-week-old specific pathogen-free C57BL/6J male mice (Jiangsu Laboratory Animal Centre, Jiangsu, China) were used in this experiment. All of the mice were housed in a barrier environment with controlled temperature (22 ± 1 °C) and humidity (55 ± 10%) under a 12 h/12 h light–dark cycle, and with free access to food and water. The experimental period was 4 weeks, and the experimental procedure is shown in Figure 1. The mice were subjected to a 7-day acclimation period (−d6 to d0) before the study. All protocols were approved by the Ethics Committee of Jiangnan University, China (qualifying number: JN. No20170803-20170912 (104), 20 July 2017), and the procedures were conducted in accordance with the European Community Guidelines for the Care and Use of Experimental Animals (Directive 2010/63/EU, 22 September 2010).

Eighteen mice were randomly divided into three groups (*n* = 6, 3 cages for each group): a control group, which was treated with normal saline (NS) daily; a JCM 1132 group, which was treated with *L. acidophilus* JCM 1132 daily; and a CCFM 720 group, which was treated with *L. acidophilus* CCFM 720 daily. Mice in each group were housed in three cages (two mice per cage) to avoid cage effect. All nine cages were close but kept isolated from each other. *L. acidophilus* (1 × 10^9^ CFU) was administered daily (once a day) to each mouse via gavage at a volume of 200 μL from d1 to d21. Mice in the control group were administered an equal volume of normal saline from d1 to d21. After 3 weeks of gavage (d1–d21, once a day), treatment was withdrawn for 1 week (d22–d28). During this withdrawal period, no bacteria or normal saline treatment was performed on the mice. Faecal samples were collected from each mouse on days 21 and 28 and stored at −80 °C prior to microbial analysis. The mice were also weighed every week. At the end of the experiment, the mice were anaesthetised and euthanized.

### 2.7. Histomorphological Analysis

Colon tissues were fixed in paraformaldehyde and dehydrated at room temperature prior to embedding in paraffin. Tissue sections (5-μm thickness) were stained with haematoxylin and eosin and scanned automatically using a Panoramic MIDI device (3D HISTECH, Budapest, Hungary).

### 2.8. Biochemical Measurements

The blood samples were allowed to clot for 2 h at room temperature prior to centrifugation for 15 min at 1000× *g*. Next, the supernatants were collected to measure serum-related parameters. Serum cytokine concentrations were detected using a Luminex MAGPIX system (Luminex, Austin, TX, USA). All of the serum samples were treated with a Milliplex MAP Kit (Merck, Germany) according to the manufacturer’s protocol prior to all cytokine assays. The concentrations of high-density lipoprotein cholesterol (HDL-C), low-density lipoprotein cholesterol (LDL-C), total cholesterol (TC), triacylglycerols (TG) and C-reactive protein (CRP) were analyzed with an automatic biochemistry meter (SELECRTA-E, Vital Scientific, Van Rensselaerweg, The Netherlands).

### 2.9. Flow Cytometry

Single-cell suspensions of the splenic tissues were prepared immediately after the mice were euthanized, according to previously reported methods [28]. The spleen cells were stained using the eBioscience Mouse Treg Staining Kit #1 (Invitrogen Corporation, Carlsbad, CA, USA) according to the manufacturer’s instructions and analysed using an Attune^®^ Nxt flow cytometer (ThermoFisher Scientific, Waltham, MA, USA).

### 2.10. 16S rDNA Amplicon Sequencing

DNA was extracted from the stool samples using a Fast DNA Stool Kit (MP Biomedicals, Santa Ana, CA, USA). The V3–V4 regions of bacterial 16S ribosomal RNA (rRNA) genes were amplified via PCR using barcode-indexed primers (341F and 806R). The products were purified by gel extraction (TIANgel Mini Purification Kit, TIANGEN, Beijing, China) and pooled in equimolar concentrations. Paired-end sequencing was performed on the Illumina MiSeq PE300 platform (Illumina, San Diego, CA, USA).

### 2.11. Determination of SCFAs in Mice Faeces

Fifty mg of fresh stool samples were homogenised in 500 μL of saturated NaCl solution and acidified with 40 μL of 10% sulphuric acid. Added to the samples was 1 mL diethyl ether to extract SCFAs, after which the samples were centrifuged at 14,000× *g* for 15 min at 4 °C. One microlitre of each supernatant was injected into an Rtx-WAX capillary column for gas chromatography-mass spectrometry (GC-MS) analysis (TSQ 9000, Thermo Scientific). The initial oven temperature (100 °C) was elevated to 140 °C at a rate of 7.5 °C/min. Then, the temperature was further elevated to 200 °C at a rate of 60 °C/min and held for 3 min. Helium was used as the carrier gas (flow rate: 0.89 mL/min, column head pressure: 62.7 kPa. The injector temperature was set at 240 °C. The mass spectrometer was set at an ion source temperature of 220 °C, an interface temperature of 250 °C and a scan range of 2–100 m/z.

### 2.12. Statistical Analysis

All of the statistical analyses in this study were performed with GraphPad Prism version 6.01 (GraphPad Software Inc., San Diego, CA, USA), SPSS 19.0 (SPSS Inc., Chicago, IL, USA), Origin 9.0.0 (OriginLab Corp., Northampton, MA, USA) and FlowJo version 10.0 (FlowJo LLC, Ashland, OR, USA). All of the values in the tables and figures are expressed as means ± standard deviations (SD). The differences between the mean values of the groups were analysed using a one-way analysis of variance with Duncan’s multiple range tests or the Kruskal–Wallis test for non-parametric analyses. The microbial data were analysed using QIIME software (GitHub, Inc., San Francisco, CA, USA). The criterion for significance was set at a *p* value < 0.05 for all comparisons.

## 3. Results

### 3.1. Loss of CCFM 720 Antimicrobial Activity against L. delbrueckii subsp. lactis

**Figure 2:**
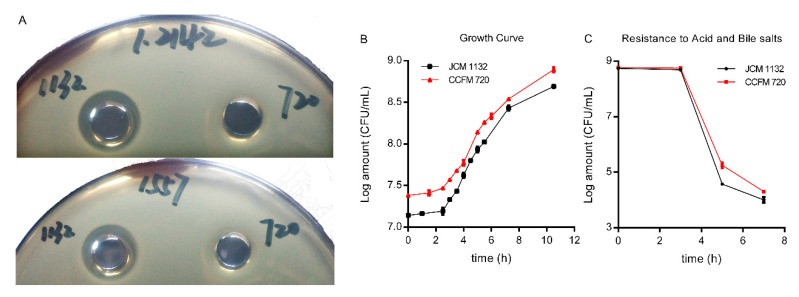
Inhibitory activity, growth curves and resistance to gastric acid and bile salts of the two strains. (**A**) Inhibition zones formed by the cell-free supernatants (CFS) produced by *L. acidophilus* JCM 1132 and CCFM 720. The indicator strains were *L. delbrueckii* subsp. *lactis* CGMCC 1.2142 and *L. delbrueckii* subsp. *lactis* JCM 1557. (**B**) Growth curves of *L. acidophilus* JCM 1132 and CCFM 720. (**C**) Capacities of acid and bile salt resistance of JCM 1132 and CCFM 720. The results are expressed as means ± standard deviations.

Two gram-negative bacteria which can infect mice and induce inflammation, and 22 g-positive bacteria were used as indicator strains to test the antibacterial activity spectra of CFS in the two strains [29]. Only the CFS produced by JCM 1132 exhibited antibacterial activity, although the observed antibacterial spectrum was narrow and included inhibitory activity only against *L. delbrueckii* subsp. *lactis* JCM 1557 and CGMCC 1.2142. The mutant strain CCFM 720 did not exhibit inhibitory activity against those indicator strains (Table 2 and Figure 2A).

### 3.2. Absence of Antimicrobial Activity in CCFM720 Was Due to the Loss of Bacteriocin Activity

We observed that the antibacterial substance in the CFS produced by JCM 1132 was less sensitive to catalase and that the size of the inhibition zone was unchanged. However, the substance was strongly sensitive to three proteases: pepsin, trypsin and papain (Table 3). As shown in Table 4, the antimicrobial substance in the CFS produced by JCM 1132 was heat-resistant, indicating that the associated activity was likely due to a peptidic compound. This result suggested that the bacteriocin produced by JCM 1132 was fairly stable at high temperatures [30]. Therefore, we believed that the absence of antibacterial activity in the CCFM 720 strain was mainly due to the loss of bacteriocin activity.

**Figure 3:**
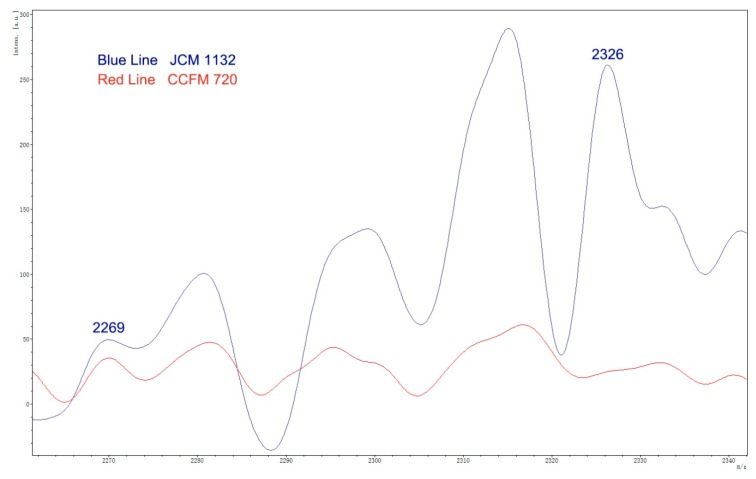
MS spectra of CFS fractions showing putative masses for α peptide (2269 Da) and β peptide (2326 Da) of Acidocin J1132.

For further confirmation, CFS supernatants of both JCM 1132 and CCFM 720 were analyzed by MALDI-TOF MS to identify the potential 2269 Da mass of the α peptide and the 2326 Da mass of the β peptide (Figure 3). A peak corresponding to a mass of 2326 Da, putative β peptide of Acidocin J1132, was identified in the CFS of JCM 1132 but not in the CFS of CCFM 720.

### 3.3. Genome Analysis

BAGEL4 was used to analyse the genomes of JCM 1132 and CCFM 720 and identify the presence of bacteriocin operons. In both cases, BAGEL4 identified the presence of structural genes corresponding to two enterolysins A (100% amino acid identity, 2 × 10^−160^), helveticin J (100% amino acid identity, 0.00) and Acidocin J1132 (100% amino acid identity, 3 × 10^−13^). The class III heat-labile bacteriocins enterolysin A and helveticin J have molecular weights of 34 and 37 kDa, respectively. The bacteriocin operons were compared at the nucleotide and amino acid levels using blastn and blastp. The enterolysin A and helveticin J operons exhibited 100% identity at both the nucleotide and amino acid levels, while the Acidocin J1132 operon exhibited 99.98% nucleotide identity and 87.46% amino acid identity. These differences can be linked to the loss of antimicrobial activity previously observed in the analysis of heat-stable antimicrobial activity, suggesting that the antimicrobial activity in JCM 1132 is attributable to intact Acidocin J1132. Proteome comparison was performed to identify the total number of different putative proteins between JCM 1132 and CCFM 720, identifying four missing putative proteins in the genome of CCFM 720, all annotated as “hypothetical proteins”, being one of them in the Acidocin J1132 cluster.

### 3.4. JCM 1132 and CCFM 720 Exhibited Similar Growth, Adhesion Characteristics and Resistance to Gastric acid and Bile Salts

The growth curves, generation times and adherence index to the Caco-2 cell line of the two strains were very similar (Figure 2B and Table 5). Furthermore, the levels of resistance to acid and bile salts were similar in both strains (Figure 2C). Based on these results, we determined that JCM 1132 and CCFM 720 behaved similarly with respect to all measured growth characteristics except bacteriocin production.

### 3.5. JCM 1132 and CCFM 720 Had Different Physiological Effects on Healthy Mice

**Figure 4:**
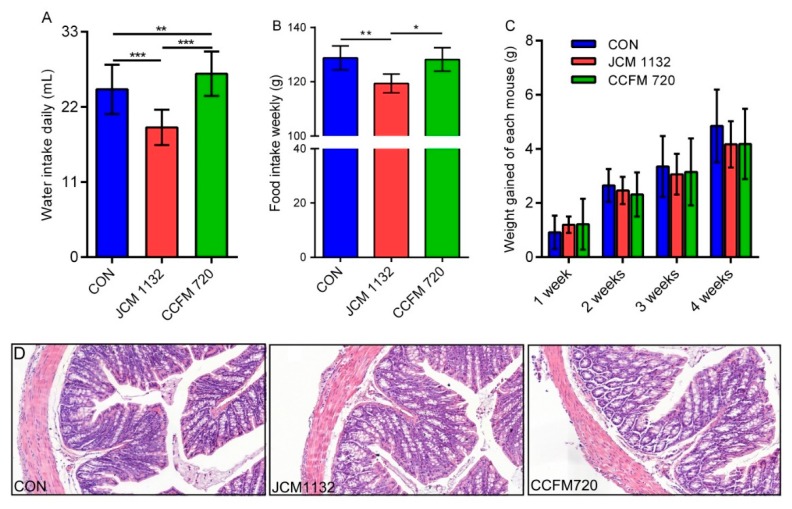
Physiological effects and morphological observations of colon tissues on different groups of mice gavaged with *L. acidophilus* JCM 1132 and CCFM 720. (**A**) Daily water intakes of different groups of mice. (**B**) Weekly food intakes of different groups of mice. (**C**) Changes in the bodyweight of each mouse in various groups. The results are expressed as means ± standard deviations. * *p* < 0.05, ** *p* < 0.01, *** *p* < 0.001. (**D**) Morphological observations of colon tissues from different groups of mice. CON, control group. JCM 1132, group gavaged with *L. acidophilus* JCM 1132. CCFM 720, group gavaged with *L. acidophilus* CCFM 720. *n* = 6 per group. Slice magnification: 200×.

The mice gavaged with JCM 1132 in all cages had significantly lower levels of daily water ingestion and weekly food intake than mice in the control group and those gavaged with CCFM 720 (Figure 4A,B). However, there was no significant difference in body weight gain among the different groups (Figure 4C). No individuals with a significant decrease in body weight gain due to reduced water and food intake were found in the JCM1132 treatment group. Moreover, no pathological changes in the colonic tissues were observed between groups gavaged with *L. acidophilus* JCM 1132 and CCFM 720 relative to the control group (Figure 4D). Therefore, our results confirm previous designations of *L. acidophilus* as GRAS.

**Figure 5:**
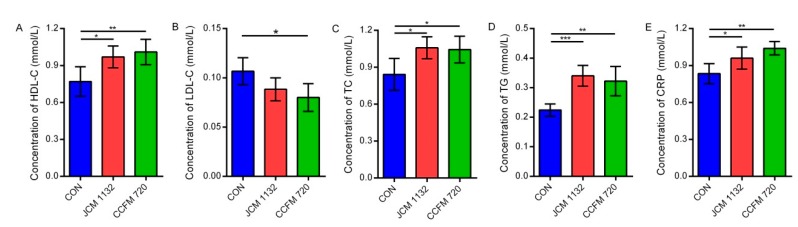
Concentrations of biochemical factors in serum samples from different groups of mice. (**A**) Concentration of HDL-C. (**B**) Concentration of LDL-C. (**C**) Concentration of TC. (**D**) Concentration of TG. (**E**) Concentration of CRP. CON, control group. JCM 1132, group gavaged with *L. acidophilus* JCM 1132. CCFM 720, group gavaged with *L. acidophilus* CCFM 720. *n* = 6 for each group. The results are expressed as means ± standard deviations. * *p* < 0.05, ** *p* < 0.01, *** *p* < 0.001. HDL-C, high-density lipoprotein cholesterol; LDL-C, low-density lipoprotein cholesterol; TC, total cholesterol; TG, triglycerides; CRP, C-reactive protein.

An analysis of serum biochemical indices showed that both JCM 1132 and CCFM 720 significantly increased the concentration of HDL-C (*p* < 0.05 and *p* < 0.01, respectively, Figure 5A) while reducing the concentration of LDL-C, although the latter was only significant in the CCFM 720 group (*p* < 0.05, Figure 5B). Both the JCM 1132 and CCFM 720 groups exhibited significant increases in the serum TC, TG and CRP concentrations relative to the control group (*p* < 0.05, Figure 5C–E).

### 3.6. JCM1132 More Effectively Promoted an Immunosuppressive Response in Healthy Mice

**Figure 6:**
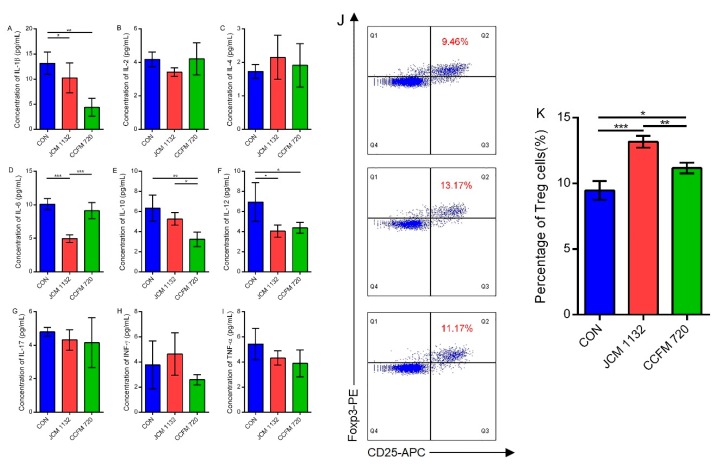
Immunological effects on different groups of mice caused by two strains respectively. (**A**–**I**) Concentrations of cytokines in serum samples from different groups of mice. CON, control group. JCM 1132, group gavaged with *L. acidophilus* JCM 1132. CCFM 720, group gavaged with *L. acidophilus* CCFM 720. n = 6 for each group. The results are expressed as means ± standard deviations. * *p* < 0.05, ** *p* < 0.01, *** *p* < 0.001. (**J**,**K**) Flow cytometry analysis of splenic CD4 + CD25 + Foxp3 + regulatory T (Treg) cells from different groups of mice. The measured splenic Treg cells are expressed as percentages of the total splenic CD4+ T lymphocytes. CON, control group. JCM 1132, group gavaged with *L. acidophilus* JCM 1132. CCFM 720, group gavaged with *L. acidophilus* CCFM 720. *n* = 6 per group. The results are expressed as means ± standard deviations. * *p* < 0.05, ** *p* < 0.01, *** *p* < 0.001.

Treatment with *L. acidophilus* JCM 1132 or CCFM 720 significantly influenced the levels of some cytokines involved in Th1, Th2 and Th17 responses in mice. Both strains significantly reduced the serum concentrations of the pro-inflammatory cytokines IL-1β and IL-12 relative to the control group (*p* < 0.05, Figure 6A,F), although CCFM 720 treatment more effectively reduced IL-1β. However, only the bacteriocin-producing strain effectively reduced the concentration of the pro-inflammatory cytokine IL-6, compared to the non-bacteriocin-producing strain (*p* < 0.001, Figure 6D). Meanwhile, only the CCFM 720 group exhibited a significant decrease in the serum concentration of the anti-inflammatory factor IL-10 (*p* < 0.01, Figure 6E). Neither strain had a significant effect on the regulation of other cytokines, such as IL-2, IL-4, IL-17, INF-γ and TNF-α, of which levels were very low but still within the detection limit (Figure 6B,C,G–I), indicating the low immunogenicity of two strains. In summary, although treatment with either JCM 1132 or CCFM 720 reduced the inflammatory response in healthy mice, JCM 1132 was more effective in this regard. All of the above indicated that *L. acidophilus* showed the potential in alleviating inflammatory response, in spite of no significant immune response induced.

As the intake of *L. acidophilus* JCM 1132 and CCFM 720 may affect the host immune system, we further studied the effects of these two strains on the populations of CD4+CD25+Foxp3+ regulatory T cells (Treg cells) in mice. Although an analysis of Treg cells from intestinal lamina propria failed because of the low abundance of cells, significant differences of Treg cell abundance among the three groups could still be found in mice spleens. Here, the percentages of Treg cells in the *L. acidophilus* JCM 1132 (13.17%) and CCFM 720 groups (11.17%) were significantly higher than those in the control group (9.46%; *p* < 0.001 and *p* < 0.05, respectively, Figure 6J). The difference between the groups treated with bacteriocin-producing and non-producing strains was also significant. JCM1132 more effectively promoted the immunosuppressive response in healthy mice (Figure 6K) which showed a correlation with the results from cytokines.

### 3.7. JCM 1132 and CCFM 720 Had Different Effects on the β Diversity of Faecal Microbiota

**Figure 7:**
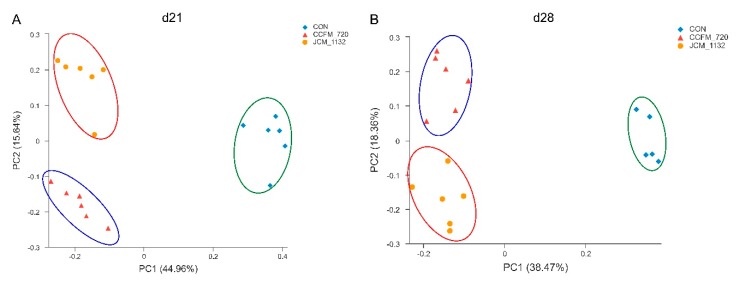
Principal coordinate analysis (PCoA) plot based on a Bray–Curtis analysis. (**A**) Day 21. (**B**) Day 28. ◆: control; ●: JCM 1132; ▲: CCFM720. Each coloured symbol represents the composition of the faecal microbiota in a single mouse; *n* = 6 per group. β diversity in the microbial communities from different groups of mice on day 21. β diversity in the microbial communities from different groups of mice on day 28.

The α diversity of faecal microbiota in mice on days 21 (gavage for 3 weeks) and 28 (withdrawal for 1 week) did not differ significantly between the groups gavaged with JCM 1132 or CCFM 720 and the control group with respect to the Simpson_1-D, Shannon_H and Chao-1 indices (Table 6). In other words, gavage with these two strains did not affect the α diversity of faecal microbiota in the mice. The β diversity of faecal microbiota is shown in Figure 7. Significant differences were detected among the two treated groups and the control group on days 21 and 28.

### 3.8. Treatment with JCM 1132 and CCFM 720 Affected Gut Microbiota Patterns at the Phylum Level

**Figure 8:**
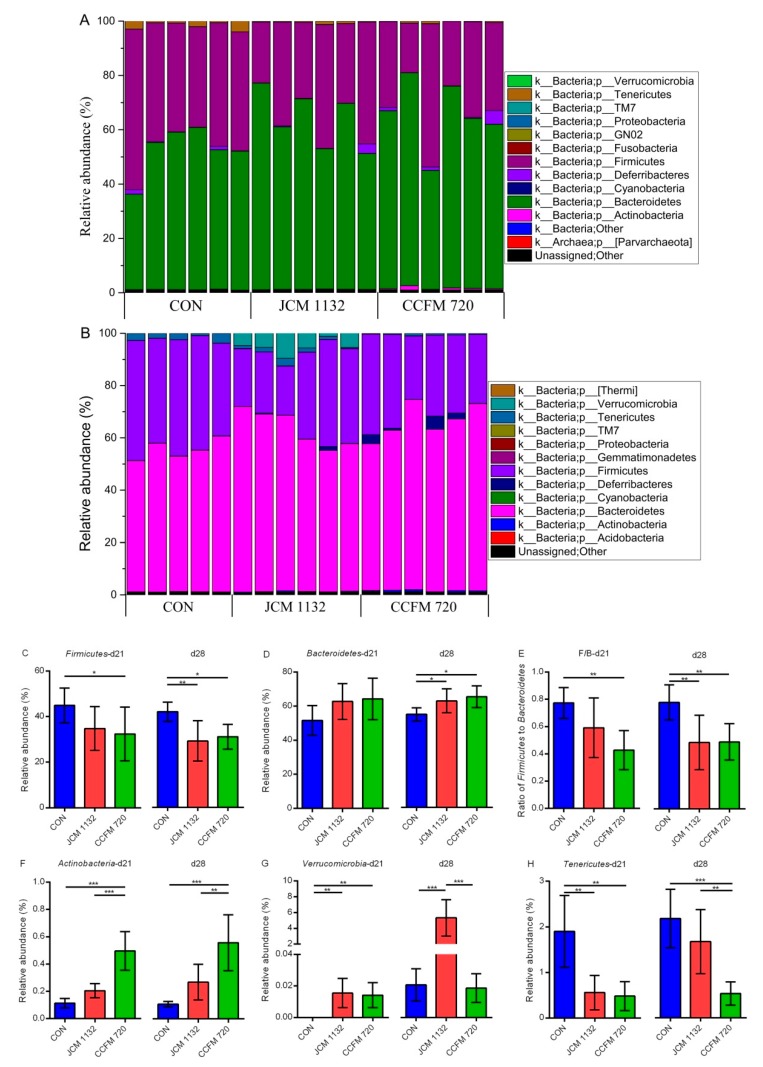
Phylum-level changes in the faecal microbiota in different groups of mice on days 21 and 28. Changes after gavage for 3 weeks (day 21). Changes after a 1-week withdrawal period (day 28). (**A**,**B**) The relative abundance of the main phyla (>0.1%) in different groups. CON, control group. JCM 1132, group gavaged with *L. acidophilus* JCM 1132. CCFM 720, group gavaged with *L. acidophilus* CCFM 720. *n* = 6 per group. (**C**–**H**) Changes in the levels of main phyla and the Firmicutes/Bacteroidetes ratio in the faecal microbiota of mice on days 21 and 28. CON, control group. JCM 1132, group gavaged with *L. acidophilus* JCM 1132. CCFM 720, group gavaged with *L. acidophilus* CCFM 720. *n* = 6 for each group. The results are expressed as means ± standard deviations. * *p* < 0.05, ** *p* < 0.01, *** *p* < 0.001.

The faecal microbiota measured on days 21 (Figure 8A) and 28 (Figure 8B) were mainly composed of six phyla (sum of relative abundance <95%), including Firmicutes, Bacteroidetes, Actinobacteria, Proteobacteria, Tenericutes and Verrucomicrobia. Of these, Firmicutes and Bacteroidetes accounted for the largest proportions. More detailed descriptions of changes in the phyla of different groups are presented below (Figure 8).

Firmicutes: The relative abundance of Firmicutes in the faecal microbiota was lower in both the JCM 1132 and CCFM 720 groups than in the control group. Compared to the control group, the proportion of this phyla was significantly lower in the CCFM 720 group on days 21 and 28 and in the JCM 1132 group on day 28. However, there was no significant difference between the JCM 1132 and CCFM 720 groups at either time point.

Bacteroidetes: The relative abundance of Bacteroidetes in the faecal microbiota tended to increase in both the JCM 1132 and CCFM 720 groups during the gavage period. No significant difference relative to control was observed until day 28 (1-week withdrawal).

Actinobacteria: Although both treated groups had a much lower relative abundance of Actinobacteria in the faecal microbiota, this abundance was significantly higher in the CCFM 720 group than in the control (*p* < 0.001) and JCM 1132 groups (*p* < 0.01).

Verrucomicrobia: All three groups had a very low (<0.03%) relative abundance of Verrucomicrobia in the faecal microflora on day 21. However, this level increased considerably in the JCM 1132 group, reaching >3% on day 28, which was significantly higher than the relative abundance in the control and CCFM 720 groups (*p* < 0.001).

Tenericutes: The relative abundance of Tenericutes was significantly higher in the control group than in the JCM 1132 and CCFM 720 groups (*p* < 0.01) on day 21. However, the relative abundance increased considerably on day 28 in the JCM 1132 group but not in the CCFM 720 group, such that the abundance in the JCM 1132 and control groups did not differ significantly and both were significantly higher than the abundance in the CCFM 720 group (*p* < 0.01).

### 3.9. Treatment with JCM 1132 and CCFM 720 Affected the Gut Microbiota Patterns at the Genus Level

**Figure 9:**
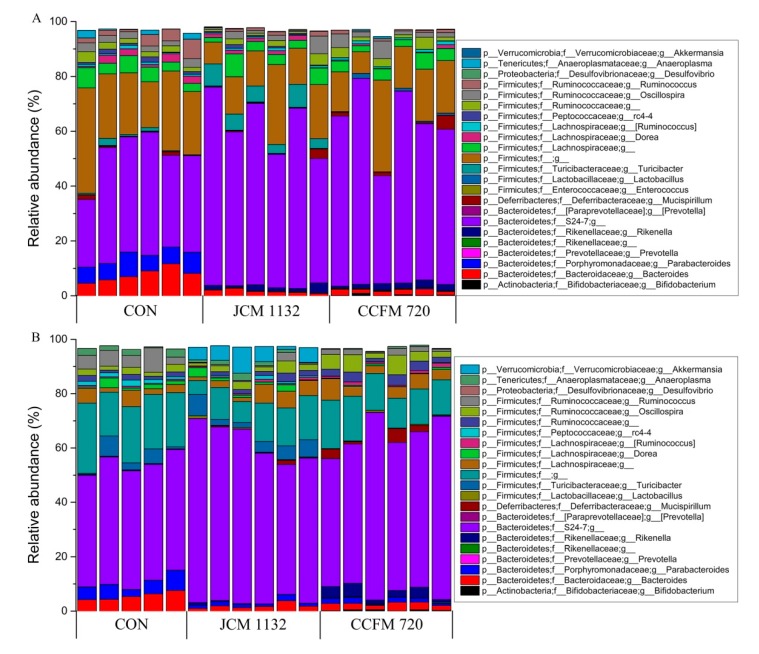

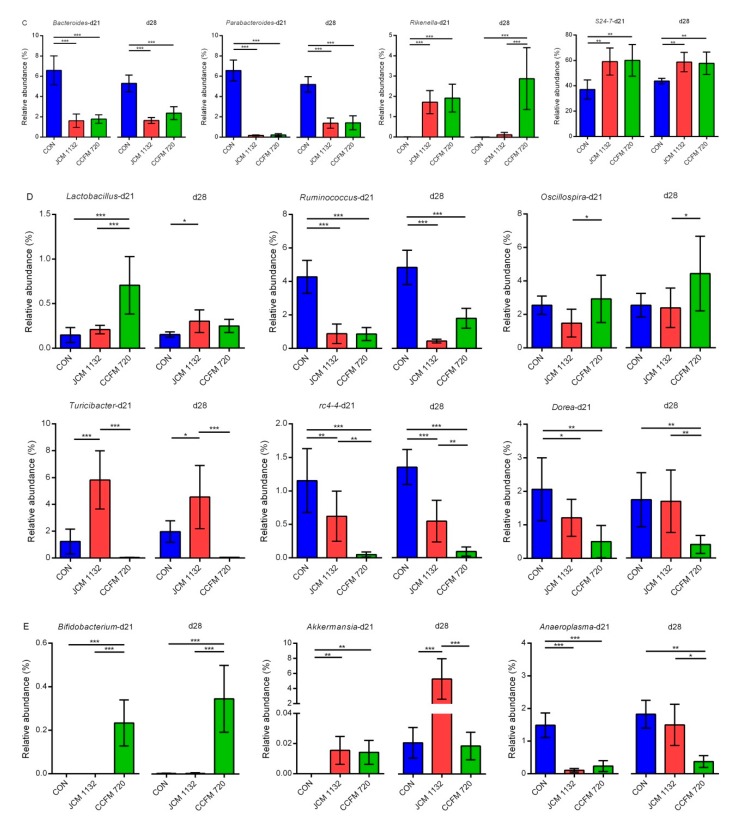
Genus-level changes in the faecal microbiota in different groups of mice on days 21 and 28. Changes after gavage for 3 weeks (day 21). Changes after a 1-week withdrawal period (day 28). (**A**,**B**) The relative abundance of the main genera (>0.1%) in different groups. CON, control group. JCM 1132, group gavaged with *L. acidophilus* JCM 1132. CCFM 720, group gavaged with *L. acidophilus* CCFM 720. *n* = 6 per group. (**C**–**E**) Changes in the levels of main genera in the faecal microbiota of mice on days 21 and 28. CON, control group. JCM 1132, group gavaged with *L. acidophilus* JCM 1132. CCFM 720, group gavaged with *L. acidophilus* CCFM 720. *n* = 6 per group. The results are expressed as means ± standard deviations. * *p* < 0.05, ** *p* < 0.01, *** *p* < 0.001.

As the oral administration of *L. acidophilus* JCM 1132 and CCFM 720 had different effects on the relative abundance of several phyla in the faecal microbiota of mice, we further analysed the microbiota at the genus level on days 21 (Figure 9A) and 28 (Figure 9B). Two unknown genera of Firmicutes and Bacteroidetes, accounted for the largest proportions (sum >50%), followed by Bacteroides, Parabacteroides, Turicibacter, Ruminococcus, Dorea and rc4-4. We observed that the oral administration of *L. acidophilus* induced changes in the levels of certain genera, and conducted a further analysis accordingly.

#### 3.9.1. Relative Abundance of Selected Genera in Bacteroidetes

The relative abundance of *Bacteroides* was significantly higher in the *L. acidophilus*-treated groups than in the control group. This increase was attributed to the level of S24-7, the genus exhibiting the greatest proportional increase not due to reduced levels of *Bacteroides* and *Parabacteroides*. Additionally, the relative abundance of *Rikenella* differed between mice treated with bacteriocin-producing and non-producing *L. acidophilus* strains (Figure 9C).

*Bacteroides*: The relative abundance of *Bacteroides* in the JCM 1132 and CCFM 720 groups on days 21 and 28 was significantly lower than that in the control group (*p* < 0.001). In other words, both strains of *L. acidophilus* appeared to have strong effects on this genus in the faecal microbiota of mice.

*Parabacteroides*: Similar to the results observed for *Bacteroides*, the relative abundance of *Parabacteroides* in the JCM 1132 and CCFM 720 groups on days 21 and 28 was significantly lower than that in the control group (*p* < 0.001), despite increases in both treated groups from days 21 to 28.

*Rikenella*: The very low relative abundance of *Rikenella* (almost 0%) in the control group was significantly lower than the corresponding values in the JCM 1132 (*p* < 0.001) and CCFM 720 groups (*p* < 0.001) on day 21. However, the relative abundance in the JCM 1132 group had decreased drastically by day 28 and was significantly lower than the abundance in the CCFM 720 group (*p* < 0.001).

*S24-7*: The relative abundance of *S24-7* in the control group was significantly lower than the values in the JCM 1132 (*p* < 0.01) and CCFM 720 groups (*p* < 0.01) at both time points.

#### 3.9.2. Relative Abundance of Selected Genus in Firmicutes

The significant decreases in the relative abundance of Firmicutes in the *L. acidophilus*-treated groups vs. the control group were attributed to reduced levels of an unknown genus (data not shown), *Ruminococcus*, *rc4-4* and *Dorea* in the former, rather than increases in the relative abundance of *Lactobacillus* and *Turicibacter* (in the bacteriocin-producing group). Moreover, the relative abundance of several genera differed between the groups treated with bacteriocin-producing and non-producing *L. acidophilus* (Figure 9D).

*Lactobacillus*: The relative abundance of *Lactobacillus* in the CCFM 720 group was significantly higher than that in the control (*p* < 0.001) and JCM 1132 groups (*p* < 0.001). However, the level of this genus in the CCFM 720 group decreased on day 28 but increased slightly in the JCM 1132 group. Although there was no significant difference between the JCM 1132 and CCFM 720 groups on days 28, the relative abundance in the JCM 1132 group was significantly higher than that in the control group (*p* < 0.05).

*Ruminococcus*: The relative abundance of *Ruminococcus* in the control group was significantly higher than those in the JCM 1132 (*p* < 0.001) and CCFM 720 groups (*p* < 0.01) on days 21 and 28.

*Oscillospira*: The relative abundance of *Oscillospira* was significantly higher in the CCFM 720 group than in the JCM 1132 group on days 21 and 28 (*p* < 0.05), despite a slight increase in the level in the JCM 1132 group at the latter time point.

*Turicibacter*: The relative abundance of *Turicibacter* was highest in the JCM 1132 group (4–6%) at both time points and was significantly higher than the levels in the CCFM 720 (*p* < 0.001) and control groups (*p* < 0.05).

*rc4-4*: The relative abundance of *rc4-4* was highest in the control group at both time points and was significantly higher than in both the JCM 1132 (*p* < 0.01) and CCFM 720 groups (*p* < 0.001). Furthermore, the relative abundance of *rc4-4* in the JCM 1132 group was significantly higher than that in the CCFM 720 group (*p* < 0.01).

*Dorea*: The relative abundance of *Dorea* was significantly higher in the control group than in the JCM 1132 (*p* < 0.05) and CCFM 720 groups (*p* < 0.01) on day 21. However, the relative abundance increased in the JCM 1132 group on day 28, at which time the level in the CCFM 720 group was significantly lower than that in the other two groups (*p* < 0.01).

#### 3.9.3. Relative Abundance of Other Genera

Bifidobacterium: The relative abundance of Bifidobacterium in the control and JCM 1132 groups on days 21 and 28 were almost 0%. In contrast, the levels in the CCFM 720 group reached 0.2–0.4% and were significantly higher than those in the other two groups (*p* < 0.001, Figure 9E).

Akkermansia: All three groups exhibited a much lower relative abundance of Akkermansia on day 21, although the levels in the JCM 1132 and CCFM 720 groups were significantly higher than those in the control group (*p* < 0.01, Figure 9E). On day 28, the relative abundance in the JCM 1132 group increased considerably to 4–6%, which was significantly higher than the levels in the control (*p* < 0.001) and CCFM 720 groups (*p* < 0.001, Figure 9E).

*Anaeroplasma*: The relative abundance of *Anaeroplasma* in the *L. acidophilus*-treated groups was significantly lower than that in the control group on day 21 (*p* < 0.001). By day 28, however, the relative abundance of this genus had increased significantly in the JCM 1132 group, whereas the level in the CCFM 720 group remained almost unchanged. Therefore, the relative abundance in the CCFM group was significantly lower than that in the JCM 1132 (*p* < 0.05) and control groups (*p* < 0.01, Figure 9E).

### 3.10. Administration of JCM 1132 and CCFM 720 Influenced the Levels and Patterns of SCFAs

We also observed different effects of CCFM720 and JCM1132 on SCFAs, consistent with the observed influences on the gut microbiota.

**Figure 10:**
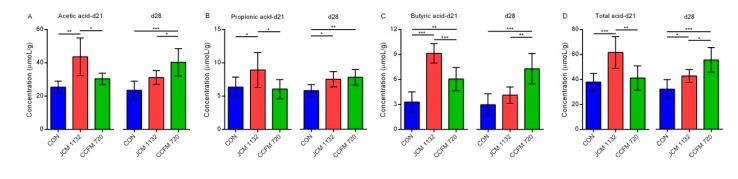
Concentrations of short-chain fatty acids (SCFAs) in the faeces from different groups of mice on days 21 and 28. (**A**) Acetic acid. (**B**) Propionic acid. (**C**) Butyric acid. (**D**) Total acid. CON, control group. JCM 1132, group gavaged with *L. acidophilus* JCM 1132. CCFM 720, group gavaged with *L. acidophilus* CCFM 720. *n* = 6 per group. The results are expressed as means ± standard deviations. * *p* < 0.05, ** *p* < 0.01, *** *p* < 0.001.

Acetic acid: The concentration of acetic acid in the JCM 1132 group was highest on day 21 and was significantly higher than the concentrations in the control (*p* < 0.01) and CCFM 720 groups (*p* < 0.05). There was no significant difference between the latter two groups at this time point. On day 28, however, the acetic acid concentration decreased significantly in the JCM 1132 group but increased significantly in the CCFM 720 group. Therefore, the concentration in the CCFM 720 group was highest on day 28 and significantly higher than the concentrations in the JCM 1132 (*p* < 0.05) and control groups (*p* < 0.001). There was no significant difference between the latter two groups at this time point (Figure 10A).

Propionic acid: On day 21, the concentration of propionic acid was highest in the JCM 1132 group and significantly higher than the concentrations in the control (*p* < 0.05) and CCFM 720 groups (*p* < 0.05). There was no significant difference between the latter two groups at this time point. On day 28, however, an increase in the propionic acid concentration in the CCFM 720 group eliminated the significant difference between the *L. acidophilus*-treated groups. The concentrations of both treated groups were significantly higher than the control group on day 28 (*p* < 0.05, Figure 10B).

Butyric acid: Similar to the observations for acetic acid, on day 21, the highest butyric acid concentration was observed in the JCM 1132 group, and this was significantly higher than the concentrations in the control (*p* < 0.001) and CCFM 720 groups (*p* < 0.001). On day 28, however, the butyric acid concentration decreased in the JCM 1132 group but increased in the CCFM 720 group. Consequently, the butyric acid concentration was significantly higher in the CCFM 720 group than in the JCM 1132 (*p* < 0.01) and control groups (*p* < 0.001). There was no significant difference between the latter two groups on day 28 (Figure 10C).

Total acid: When we combined the concentrations of the three above-listed SCFAs, we observed that the patterns of total acid (sum of acetic acid, propionic acid and butyric acid) were similar to those of acetic acid and butyric acid. On day 21, the total acid content was significantly higher in the JCM 1132 group than in the control (*p* < 0.001) and CCFM 720 groups (*p* < 0.01). There was no significant difference between the latter two groups. On day 28, however, a decrease in the total acid content in the JCM 1132 group and an increase in the CCFM 720 group led to a significantly higher total acid content in the CCFM group relative to the control (*p* < 0.001) and JCM 1132 groups (*p* < 0.05). However, the total acid content in the JCM 1132 group remained significantly higher than that in the control group (*p* < 0.05; Figure 10D).

## 4. Discussion

Some LAB may contribute to intestinal micro-ecological homeostasis and can improve host health [31,32,33]. Bacteriocins are antimicrobial peptides produced by some LAB, which play important roles as probiotics. In recent years, increased research into LAB bacteriocins has led to the continuous development of bacteriocin-producing strains for use in food and manufacturing processes as a means of controlling microbiota communities in food systems and inhibiting the growth of pathogenic bacteria [34,35]. Moreover, these bacteriocin-producing LAB have been proposed as alternatives to antibiotics for the treatment of infections caused by pathogenic bacteria, including some drug-resistant bacteria. These LAB have a narrow spectrum and thus have little effect on the host gut microbiota [36,37]. However, few studies have evaluated the effects of bacteriocin-producing LAB on the healthy gut microbiota and metabolite composition. Only a few studies have mentioned the transient effects of LAB bacteriocin on the gut microbiota. For instance, neither Abp118-producing nor non-producing strains of *L. salivarius* had significant effects on the gut microbiota at the phylum level in the pig and mouse, other than a significant decrease in *Spirochaete* [18]. Plantaricin, which is produced by *L. plantarum* P-8, also induced a shift in the human faecal bacterial structure [19]. In this study, we evaluate the effects of the bacteriocin-producing *L. acidophilus* strain JCM 1132 and its bacteriocin-free spontaneous mutant CCFM 720 on the gut microbiota and metabolism in healthy mice. Although these strains have not previously been reported to have significant effects on body weight [18,38], the bacteriocin-producing *L. acidophilus* strain significantly affected the food and water intake of the mice in our study, as well as some physiological and immunological indicators. An analysis of the gut microbiota revealed that treatment with *L. acidophilus* JCM 1132 and CCFM 720 induced significant differences in the levels of several genera in the gut microbiota of healthy mice, as well as changes in SCFA production.

The bacteriocins synthesized by LAB can be roughly divided into three categories: Class I, Class II and Class III [39,40,41]. The bacteriocin identified by BAGEL4 in the *L. acidophilus* JCM 1132 genome was Acidocin J1132, a well-known pore-forming bacteriocin. Acidocin J1132 is a two-component Class IIb bacteriocin, formed by a subunit α and β. Acidocin J1132 has a narrow antimicrobial spectrum, with inhibitory effects only against some species of lactobacilli [30]. Although the effect of Acidocin J1132 on the gut microbiota has not been studied, the effects of other Class II bacteriocins on the gut microbiota have been reported. In a previous study, five Class II bacteriocins with different antimicrobial spectra had little effect on the gut microbiota at the phylum level, whereas wide-spectrum bacteriocins induced transient changes at the genus level which were generally considered to be beneficial [42]. In our study, despite the narrow antibacterial spectrum of bacteriocin from JCM 1132, the different effects of the *L. acidophilus* JCM 1132 and CCFM 720 strains on certain genera in the gut microbiota persisted even one week after gavage withdrawal. Therefore, the effects of these LAB on gut microbiota may be more persistent than expected, possibly because of the formation of a new steady-state via competition within the gut microbiota. It is also necessary to consider the duration for which the LAB resides in the intestinal tract. For a potentially intestine-colonizing bacterium, the production of bacteriocin may determine the effect on the gut microbiota in situ. Although the generation time and adherence index to the Caco-2 cell line of two strains are similar in vitro, it is conceivable that the lack of antibacterial activity may affect the competitive growth of bacteria in the intestine and colonisation. The lack of antibacterial activity may have an all-round effect on the growth of *L. acidophilus* in the intestine. Therefore, it can not be ruled out that some of the changes may be driven by altered growth/colonisation ability, but we believe that the altered growth/colonisation ability between the two strains, if any, may be largely driven by the loss of antibacterial activity.

In general, our results indicate that neither *L. acidophilus* JCM 1132 nor CCFM 720 significantly affects the α diversity of the gut microbiota. This finding appears advantageous, as it suggests that probiotics do not induce large structural changes in the host gut microbiota. In contrast, the results of β diversity analyses revealed significant differences in the gut microbiota between the *L. acidophilus* JCM 1132 and CCFM 720 groups. These differences were confirmed by changes at the phylum level, as the two strains differently affected the abundance of Actinobacteria, Verrucomicrobia and Tenericutes, and these changes had significantly different durations. Among them, the effect of *L. acidophilus* on Actinobacteria may have been mainly due to its effect on *Bifidobacterium*. Previously, *L. acidophilus* was shown to increase the abundance of *Bifidobacterium* in BALB/c mice [43]. The presence of *Bifidobacterium*, a typical beneficial bacteria, in the host intestinal tract often indicates an improvement in the intestinal environment [44]. However, our results indicate that only treatment with *L. acidophilus* CCFM 720 led to a significant increase in and the stable maintenance of the abundance of *Bifidobacterium* in the mouse intestine. This observation suggests that the increased abundance of *Bifidobacterium* induced by *L. acidophilus* may be offset by bacteriocin. Specifically, this offset may be due not directly to the inhibition of *Bifidobacterium* by bacteriocin, but rather to feedback from the inhibition of other intestinal bacteria. The beneficial bacteria *Akkermansia* was also shown to be differentially affected by *L. acidophilus*. *A. muciniphila*, a member of Verrucomicrobia, is an important physiological regulator and next-generation probiotic that is beneficial to humans [45,46,47]. In previous studies, a close relationship was observed between the abundance of *A. muciniphila* and improved metabolic signs in mice, and supplementation with this strain could regulate high-fat diet-related glucose tolerance and relieve adipose tissue inflammation [48,49,50]. Our results showed that gavage with *L. acidophilus* JCM 1132 or CCFM 720 did not affect the slight increase in the abundance of *Akkermansia*. Interestingly, the abundance of *Akkermansia* in the JCM 1132 group increased significantly after a 1-week withdrawal period. This large increase may have been caused by a re-organization of the microbiota structure after *L. acidophilus* administration. The effect of *L. acidophilus* JCM 1132 on *Akkermansia* may also guide approaches to the alleviation of metabolic diseases such as obesity and diabetes.

The intake of *L. acidophilus* also reduced the Firmicutes/Bacteroidetes (F/B) ratio and the abundance of Tenericutes. Studies have shown that obesity might be linked to the gut microbiota composition in the host. The relative abundance of Firmicutes and Bacteroides in obese mice was shown to significantly increase and decrease, respectively, resulting in an increase in the harvesting of energy in the host [51,52]. A similar gut microbiota structure has also been observed in patients with type 2 diabetes and metabolic syndrome [53]. The abundance of Tenericutes has been linked to obesity or type 2 diabetes-associated metabolic parameters [54,55]. Although the effects of reducing the F/B ratio did not appear to be directly related to the presence or absence of bacteriocin during gavage in our study, treatment with *L. acidophilus* JCM 1132 had a delayed reducing effect on the F/B ratio. The difference in the reduction in Tenericutes abundance between the groups treated with the two *L. acidophilus* strains only manifested after a 1-week withdrawal period. Moreover, the decreased abundance of Tenericutes associated with *L. acidophilus* JCM 1132 treatment disappeared rapidly, indicating that this phenomenon might not be the result of direct inhibition by the *L. acidophilus* JCM 1132. Regarding the mechanism by which *L. acidophilus* JCM 1132 treatment affects *Akkermansia*, we can speculate that bacteriocin induces changes in the gut microbiota by killing target bacteria directly. The subsequent disappearance of bacteriocin may then cause a rapid over-correction. The ability of *L. acidophilus* to control/balance the population of beneficial gut microbiota that related to obesity, metabolic syndrome, diabetes etc. suggest that these two strains may help to maintain intestinal health and metabolic balance in the host. However, the targeted microbiota and ways of adjustment were different between these two strains, although the two strains did not seem to have significantly different effects on indicators of lipid metabolism.

Acidocin J1132 exhibits a narrow antibacterial spectrum against some *Lactobacillus* species in vitro [30]. In this study, the intrinsic *Lactobacillus* strains in the gut were inhibited and substituted by *L. acidophilus* JCM 1132 during the gavage period but rebounded slightly after a 1-week withdrawal period. This finding suggests that the administration of *L. acidophilus* JCM 1132 triggered or replaced the growth of intrinsic *Lactobacillus*, as suggested previously [56]. The rapid decrease in the abundance of *Lactobacillus* in the *L. acidophilus* CCFM 720 group also indicated that the abundance of this species was maintained within a relatively stable range in the healthy gut. *L. acidophilus* JCM 1132 does not manifest an outstanding capacity for colonization [57], although this situation may be more prominent in different host species [58]. Non-mouse-derived, non-bacteriocin-producing *L. acidophilus* strains face a disadvantage with respect to the colonisation and replacement of native lactobacilli in the mouse intestine. Even in the presence of bacteriocin, a proportion of the intrinsic lactobacilli may be killed, but the abundance of *Lactobacillus* will not change greatly. This characteristic is beneficial for maintaining homeostasis in the host gut microbiota.

Our analysis of other genera revealed that the effect of *L. acidophilus* on the relative abundance of Bacteroidetes was mainly due to an increase in the abundance of *S24-7*. However, the effects of *L. acidophilus* on *S24-7*, *Bacteroides* and *Parabacteroides* are unlikely to be related directly to the effect of bacteriocin. Previously, a combination of polysaccharide with probiotics (*L. acidophilus* NCFM and *B. lactis* Bi-07) was shown to decrease the proportion of *Parabacteroides* in gut microbiota significantly in weaned rats [59]. Antibacterial substances other than bacteriocins produced by LAB, such as organic acids and hydrogen peroxide, can also influence the intestinal environment [60,61]. However, we observed a considerable difference in the regulation of the relative *Rikenella* abundance by *L. acidophilus* between the *L. acidophilus* JCM 1132 and CCFM 720 groups after a 1-week withdrawal period. Previous studies have revealed a negative correlation between the abundance of *Rikenella* and body weight or fat mass [62,63]. This increase in the abundance of *Rikenella* also confirms the role of *L. acidophilus* in maintaining metabolic health in the host. As with the mechanism used to regulate the abundance of Tenericutes, treatment with *L. acidophilus* CCFM 720 appeared to better support a lasting beneficial effect on host metabolism.

Additionally, gavage with the different bacterial strains led to differential regulation of the abundance of the Firmicutes genera *Turicibacter*, *Oscillospira, rc4-4* and *Dorea*. In particular, *L. acidophilus* CCFM 720 more strongly inhibited *rc4-4* and *Dorea*, and had a more persistent inhibitory effect on the latter genus. Moreover, after a 1-week withdrawal period, treatment with *L. acidophilus* CCFM 720 was associated with an increase in the abundance of *Oscillospira*, whereas treatment with JCM 1132 had a stronger inhibitory effect on *Ruminococcus*. An increased abundance of *rc4-4* was shown to be associated with high-fat diet-induced obesity [64,65]. Moreover, several reports have described associations between a high abundance of *Dorea* and some diseases [66,67,68]. *Ruminococcus* is thought to be associated with inflammatory bowel disease, while *Oscillospira* has been reported to improve weight loss [69,70]. As with the F/B ratio, Tenericutes and *Rikenella*, the altered patterns of *rc4-4, Dorea*, and *Oscillospira* suggest that treatment with *L. acidophilus* CCFM 720 may have a more lasting beneficial effect on metabolism. The effects of the two *L. acidophilus* strains on the levels of beneficial SCFAs after a 1-week withdrawal period also supported this conjecture.

In contrast to the above conclusions, however, treatment with either JCM 1132 or CCFM 720 led to a decreased abundance of *Anaeroplasma*, which was reported to be correlated negatively with obesity and hypercholesterolemia [71,72]. Additionally, CCFM 720 had a more persistent inhibitory effect. In our experiments, only *L. acidophilus* JCM 1132 had a beneficial regulatory effect on the abundance of *Turicibacter* which was probably due to the reversal of the inhibitory effect of bacteriocin on *Turicibacter*. *Turicibacter* has been reported to alleviate the symptoms of hyperlipidaemia and relieve inflammation in the body [73,74]. Treatment with *L. acidophilus* JCM 1132 also more strongly inhibited species of the pro-inflammatory genus *Ruminococcus* [69]. The ability of JCM 1132 treatment to regulate the abundance of *Turicibacter* and *Ruminococcus* may also explain why this strain more effectively promoted an immunosuppressive response.

Our results suggest that *L. acidophilus* JCM 1132 and CCFM 720 have different effects on the gut microbiota of healthy mice, as well as some physiological indicators, and that some of these differences persist even one week after treatment withdrawal. Although previous studies have suggested that the differential effects of bacteria with or without bacteriocins on the gut microbiota would disappear at the end of the experimental process, our results indicate persistent effects on some phyla or genera, despite significant effects on the gut microbiota diversity. High doses of bacteriocin may pose a health risk to the host [75]. Therefore, although LAB are GRAS, our research suggests that further studies of the effects of bacteriocin associated with LAB applications are needed. However, it must be acknowledged that, because of the currently unavailable on genetic modification on *L. acidophilus* JCM 1132 or CCFM 720 in this research, there is a potential possibility that CCFM 720 is not a perfect bacteriocin non-producing control for JCM 1132. Although no obvious differences in the other physiological characteristics of the two strains have been found in addition to the loss of antibacterial ability and putative β peptide of Acidocin J1132, we cannot rule out the potential impacts of the other three missing putative proteins on the physiological characteristics of *L. acidophilus* JCM1132. The underlying factors which impacted mice gut microbiota other than bacteriocin cannot be completely ruled out, such as colonisation and metabolite production. Therefore, further attempts to genetically modify the Acidocin encoding loci in JCM1132 or complement CCFM 720 with this locus from JCM1132 will help us to more convincingly determine the presence or absence of bacteriocin on the effects by *L. acidophilus* on healthy mice. In addition, further work would be needed to assess which of the observed outcomes are due to the direct effect of different doses of the bacteriocin, once purified, and which are indirect effects linked to modifications on the gut microbiota structure.

## 5. Conclusions

In summary, we found that treatment with *L. acidophilus* JCM1132 and CCFM 720 had different effects on the gut microbiota composition and metabolite production in healthy hosts. Moreover, treatment with the latter strain appeared to have more persistent beneficial effects on host metabolism. To fully understand the value and safety of LAB with regard to host health, further studies should evaluate the effects of bacteriocin-producing and non-producing LAB on intestinal ecology in the gut environment and attempt to establish a link between bacteriocin-producing LAB and host health. Targeting the effects of bacteriocin gene-manipulated bacteria or even the enveloped purified bacteriocin on the gut microbiota and host health will be effective research methods. Our study findings provide a reference for the selection and development of probiotics in the future.

## Figures and Tables

**Table 1:** Isolates used in this study and culture conditions.

Species	Strain	Source ^a^	Culture Conditions
Tested strains			
*Lactobacillus acidophilus*	JCM 1132	JCM	37 °C, MRS, Anaerobic
	CCFM 720	CCFM	37 °C, MRS, Anaerobic
Indicator strains			
*Lactobacillus plantarum*	S8	CCFM	37 °C, MRS, Aerobic
*Lactobacillus coryniformis*	S16	CCFM	37 °C, MRS, Aerobic
*Lactobacillus plantarum*	S17	CCFM	37 °C, MRS, Aerobic
*Lactobacillus fermentum*	S40	CCFM	37 °C, MRS, Aerobic
*Lactobacillus casei*	W3	CCFM	37 °C, MRS, Aerobic
*Lactobacillus plantarum*	W6	CCFM	37 °C, MRS, Aerobic
*Lactobacillus plantarum*	W11	CCFM	37 °C, MRS, Aerobic
*Lactobacillus plantarum*	W13	CCFM	37 °C, MRS, Aerobic
*Lactobacillus plantarum*	W19	CCFM	37 °C, MRS, Aerobic
Entero-invasive *Escherichia coli*	ATCC 43893	CCFM	37 °C, TSB, Aerobic
*Staphylococcus aureus*	*S. aureus*	CCFM	37 °C, TSB, Aerobic
*Salmonella typhimurium*	SL1344	CCFM	37 °C, TSB, Aerobic
*Enterococcus faecalis*	*E. faecalis*	CCFM	37 °C, TSB, Aerobic
*Lactobacillus reuteri*	9-5	CCFM	37 °C, MRS, Aerobic
*Lactobacillus reuteri*	L103	CCFM	37 °C, MRS, Aerobic
*Lactococcus Lactis*	N5	CCFM	37 °C, MRS, Aerobic
*Lactobacillus rhamnosus*	LGG	CCFM	37 °C, MRS, Aerobic
*Lactobacillus salivarius*	ZX5	CCFM	37 °C, MRS, Aerobic
*Lactobacillus fermentum*	D2	CCFM	37 °C, MRS, Aerobic
*Lactobacillus acidophilus*	FFJND6-L5	CCFM	37 °C, MRS, Aerobic
*Lactobacillus crispatus*	FHUBES1M18	CCFM	37 °C, MRS, Aerobic
*Lactobacillus crispatus*	FHUBES1M24	CCFM	37 °C, MRS, Aerobic
*Listeria monocytogenes*	ATCC 19114	ATCC	37 °C, TSB, Aerobic
*Streptococcus mutans*	ATCC 25175	ATCC	37 °C, TSB, Aerobic
*Lactobacillus delbrueckii subsp. lactis*	JCM 1557	JCM	37 °C, MRS, Aerobic
*Lactobacillus delbrueckii subsp. lactis*	CGMCC 1.2142	CGMCC	37 °C, MRS, Aerobic
*Lactobacillus paracasei*	CICC 20241	CICC	37 °C, MRS, Aerobic
*Lactobacillus casei*	CICC 20975	CICC	37 °C, MRS, Aerobic

^a^ CCFM is an abbreviation for Culture Collection of Food Microorganisms at Jiangnan University. JCM is an abbreviation for Japan Collection of Microorganisms. ATCC is an abbreviation for American Type Culture Collection. CGMCC is an abbreviation for China General Microbiological Culture Collection Center. CICC is an abbreviation for China Center of Industrial Culture Collection.

**Table 2:** Antimicrobial ability of CFS produced by two strains.

Indicator Strains	Diameter of the Zone of Inhibition (mm) ^a,b^	
	JCM 1132	CCFM 720
Gram-negative bacteria		
ATCC 43893	8	8
SL1344	8	8
Gram-positive bacteria		
S16	8	8
S17	8	8
S40	8	8
W11	8	8
W13	8	8
W19	8	8
*S. aureus*	8	8
*E. faecalis*	8	8
9-5	8	8
L103	8	8
N5	8	8
LGG	8	8
ZX5	8	8
D2	8	8
FFJND6-L5	8	8
FHUBES1M18	8	8
FHUBES1M24	8	8
ATCC 19114	8	8
ATCC 25175	8	8
JCM 1557	11.3 ± 0.3	8
CGMCC 1.2142	10.7 ± 0.3	8
CICC 20241	8	8

^a^ The outside diameter of Oxford Cup is 8 mm. ^b^ The results are expressed as “mean ± SD”.

**Table 3:** Effects of proteases and catalase on the antimicrobial effect of CFS of 2 strains.

Treatment	Diameter of the Zone of Inhibition for CFS of JCM 1132 (mm) ^a,b^
Without Protease	11.3 ± 0.3
Catalase	11.0 ± 0.0
Pepsin	8
Trypsin	8
Papain	8

^a^ Indicator strain used for determination is *L. delbrueckii* subsp. *lactis* JCM 1557. ^b^ The outside diameter of the Oxford Cup is 8 mm and the results are expressed as “mean ± SD”.

**Table 4:** Effects of heat treatments on the antimicrobial effect of CFS of JCM 1132 and CCFM 720.

Treatment	Diameter of the Zone of Inhibition for CFS of JCM 1132 (mm) ^a,b^	Diameter of the Zone of Inhibition for CFS of CCFM 720 (mm) ^a,b^
Without Heat	11.3 ± 0.3	8
60 °C for 10 min	10.8 ± 0.2	8
60 °C for 30 min	10.5 ± 0.2	8
60 °C for 1 h	10.5 ± 0.1	8
90 °C for 10 min	10.3 ± 0.2	8
90 °C for 30 min	10.3 ± 0.1	8
121 °C for 15 min	10.3 ± 0.2	8

^a^ Indicator strain used for determination is *L. delbrueckii* subsp. *lactis* JCM 1557. ^b^ The outside diameter of Oxford Cup is 8 mm and the results are expressed as “mean ± SD”.

**Table 5:** Generation time and adherence index to the Caco-2 cell line of two strains.

Species	Strain	Generation Time	Adherence Index
*L. acidophilus*	JCM 1132	0.77	7.4 ± 0.5
	CCFM 720	0.77	8.1 ± 0.3

**Table 6:** α diversity indexes of fecal microbiota in different groups on days 21 and 28.

Group	After Gavage for 3 Weeks (d21) ^a,b^			After Withdraw for 1 Week (d28) ^a,b^		
	Simpson_1-D	Shannon_H	Chao-1	Simpson_1-D	Shannon_H	Chao-1
CON	0.96 ± 0.00	4.77 ± 0.13	15,560.50 ± 3660.38	0.95 ± 0.00	4.53 ± 0.19	16,100.00 ± 2001.66
JCM 1132	0.96 ± 0.01	4.75 ± 0.25	13,123.33 ± 727.97	0.95 ± 0.01	4.63 ± 0.26	18,068.33 ± 4413.65
CCFM 720	0.95 ± 0.02	4.81 ± 0.22	12,556.67 ± 1407.46	0.95 ± 0.01	4.72 ± 0.23	16,121.67 ± 3694.31

^a^ CON, the control group. JCM 1132, group gavaged *L. acidophilus* JCM 1132. CCFM 720, group gavaged *L. acidophilus* CCFM 720, *n* = 6 for each group. ^b^ The results are expressed as “mean ± SD”.
